# GAG-DB, the New Interface of the Three-Dimensional Landscape of Glycosaminoglycans

**DOI:** 10.3390/biom10121660

**Published:** 2020-12-11

**Authors:** Serge Pérez, François Bonnardel, Frédérique Lisacek, Anne Imberty, Sylvie Ricard Blum, Olga Makshakova

**Affiliations:** 1 University Grenoble Alpes, CNRS, CERMAV, 38000 Grenoble, France; francois.bonnardel@cermav.cnrs.fr (F.B.); anne.imberty@cermav.cnrs.fr (A.I.); 2 SIB Swiss Institute of Bioinformatics, CH-1227 Geneva, Switzerland; frederique.lisacek@sib.swiss; 3 Computer Science Department, University of Geneva, CH-1227 Geneva, Switzerland; 4 Section of Biology, University of Geneva, CH-1205 Geneva, Switzerland; 5 Institut de Chimie et Biochimie Moléculaires et Supramoléculaires, UMR 5246 CNRS—Université Lyon 1, 69622 Villeurbanne CEDEX, France; sylvie.ricard-blum@univ-lyon1.fr; 6 Kazan Institute of Biochemistry and Biophysics, FRC Kazan Scientific Center of RAS, 420111 Kazan, Russia; olga.makshakova@kibb.knc.ru

**Keywords:** glycosaminoglycans, three-dimensional structure, database, polysaccharide conformation, protein-carbohydrate interactions

## Abstract

Glycosaminoglycans (GAGs) are complex linear polysaccharides. GAG-DB is a curated database that classifies the three-dimensional features of the six mammalian GAGs (chondroitin sulfate, dermatan sulfate, heparin, heparan sulfate, hyaluronan, and keratan sulfate) and their oligosaccharides complexed with proteins. The entries are structures of GAG and GAG-protein complexes determined by X-ray single-crystal diffraction methods, X-ray fiber diffractometry, solution NMR spectroscopy, and scattering data often associated with molecular modeling. We designed the database architecture and the navigation tools to query the database with the Protein Data Bank (PDB), UniProtKB, and GlyTouCan (universal glycan repository) identifiers. Special attention was devoted to the description of the bound glycan ligands using simple graphical representation and numerical format for cross-referencing to other databases in glycoscience and functional data. GAG-DB provides detailed information on GAGs, their bound protein ligands, and features their interactions using several open access applications. Binding covers interactions between monosaccharides and protein monosaccharide units and the evaluation of quaternary structure. GAG-DB is freely available.

## 1. Introduction

Proteoglycans (PGs) constitute a diverse family of proteins that occur in the extracellular matrix (ECM) and pericellular matrix (PCM) and on the surface of mammalian cells. They consist of a core protein and one or more covalently attached glycosaminoglycan (GAG) chains. PGs play critical roles in numerous biological processes, which are mediated by both their protein part and their GAG chains [1,2].

**Figure 1:**
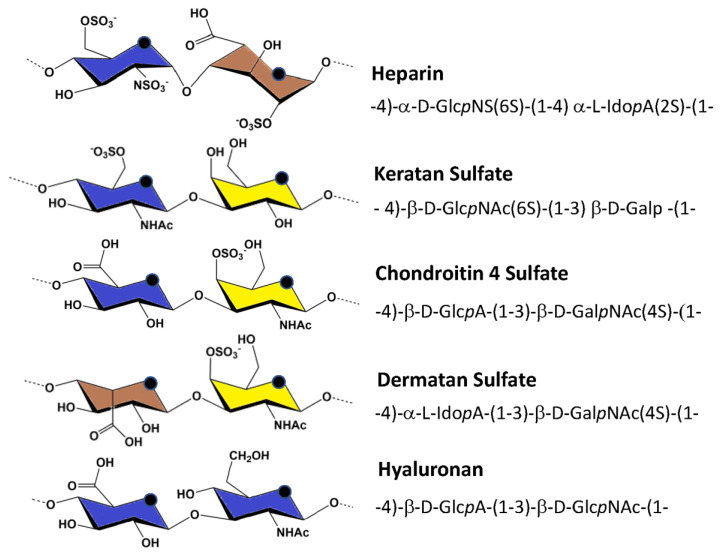
Main repeating units of glycosaminoglycans. The color-coding of the constituting monosaccharide complies with SNFG nomenclature [22]. The abbreviations are as follows: Glc*p* for glucose, Ido*p* for idose), Gal*p* for galactose, N for amine, S for sulfate, A for acid, and NAc for N-acetyl.

GAGs refer to six major polysaccharides in mammals: chondroitin sulfate (CS) [3], dermatan sulfate (DS), heparin (HP), heparan sulfate (HS) [4,5], hyaluronan (HA) [6], and keratan sulfate [7,8]. Their molecular mass ranges from a few kDa to several million Da for hyaluronan. Despite significant compositional differences, GAGs also share common features. They are linear polysaccharides made of disaccharide repeats. The disaccharides are composed of uronic acid and an hexosamine, alternatively linked through 1-4 and 1-3 glycosidic bonds (Figure 1), except for keratan sulfate, which involves galactose (Gal*p*) and N-acetylglucosamine (Glc*p*NAc) [7]. In contrast to the five other GAGs, hyaluronan is not sulfated and does not bind covalently to proteins to form proteoglycans. Variations in the pattern of GAG sulfation at various positions, create an impressive structural diversity. Two hundred and two unique disaccharides of mammalian GAGs have been identified so far, including 48 theoretical disaccharides in HS [9].

In addition to their contribution to the physicochemical properties of PGs, GAGs play an essential role in the organization and assembly of the extracellular matrix. They also regulate numerous biological processes by interacting with proteins in the extracellular milieu and at the cell surface. The six mammalian GAGs were shown to interact with 827 proteins in the recently published GAG interactome [10]. 

Many of these GAG interactions have been investigated and characterized in health and disease. According to [10], they take place in various locations (intracellular, cell surface, secreted, and blood proteins) and the protein partners range from individual growth factors (e.g., fibroblast growth factor-2) to large multidomain extracellular proteins such as collagens I and V, and fibronectin with different affinity and half-life [11,12]. These proteins are involved in a variety of biological processes such as extracellular matrix assembly, cell signaling, development, and angiogenesis [10,13,14]. Besides, glycosaminoglycans play a role in host-pathogen interactions by binding to bacterial, viral, and parasite proteins [15,16,17,18,19,20]. The significance of the understanding and mastering the molecular features underlying the interaction of GAGs to proteins was magistrally demonstrated by the development of the antithrombotic drugs as reviewed in [21].

The length, sequence, substitution pattern, charge, and shape of GAGs control both their physicochemical properties and their biological functions. Understanding the functions of GAGs first requires methods to accurately assess their molecular weight, their composition and their sequences. This is made possible through ongoing progress in mass spectrometry, and heparan sulfate has been sequenced by liquid chromatography-tandem mass spectrometry (LC-MS/MS) [23,24,25,26,27]. Furthermore, the structural and conformational complexity of GAGs challenges the characterization of their three-dimensional features using either experimental or theoretical methods. In a sense, GAGs concentrate most on the difficulties faced in structural glycoscience. They combine the challenges associated with both glycans and polyelectrolytes. Several experimental techniques have been used to solve GAG structures, including fiber X-ray crystallography, nuclear magnetic resonance (NMR) [28,29], electron microscopy, small-angle X-ray scattering (SAXS) [30], and neutron scattering (elastic incoherent neutron scattering EINS [31], and small-angle neutron scattering SANS [32]. Still, no single technique can cope with such complexity, but, computational methods offer valuable tools to integrate partial information collected experimentally. These, in turn, are useful to validate and improve simulation strategies. However, these approaches remain limited due to the intrinsic properties of GAGs. Like any other complex glycans, they are highly flexible, create many solvent-mediated interactions and have a polyanionic character. Nevertheless, progress in this field is underway, as detailed in [33] that investigates structures from monosaccharides to polysaccharides.

GAG-protein complex structures available in the PDB have been compiled by Samsonov and coworkers [34]. They concluded that this dataset does not represent the diversity of natural GAG sequences. It implies that computational approaches will be critical in understanding GAG structural biology and their mechanisms of interaction with their protein partners [35,36,37]. Significant progress has been made to investigate GAG structures, isolated and complexed with proteins, both at all-atom and coarse-grained levels [33,38,39,40,41]. However, appropriate tools for data mining of GAG-protein interactions are still missing [12,14].

MatrixDB (http://matrixdb.univ-lyon1.fr/) is a biological database focused on molecular interactions between extracellular proteins and polysaccharides [42]. It offers the first step to investigate the molecular mechanisms of GAGs-protein interactions. In this resource, building and displaying the three-dimensional structural models of GAGs was rationalized through an effort to standardize the format of GAGs sequences and group GAG disaccharides into a limited number of families [9]. However, the relative spatial orientations of key GAG chemical groups interacting with (potential) “hot spots” on the proteins was not characterized. The conformational features displayed by the long-chain GAGs polysaccharides were not considered either. To move forward, we collected further evidence of experimental GAG and GAG-protein interaction data, from databases and in the relevant literature.

Experimental details of protein or protein complex three-dimensional structures are comprehensively recorded in the Protein Data Base [43]. While being an essential repository, the glycan-related data stored in the PDB is not easily accessible to non-glycoscientists. This difficulty was identified in the glycoscience community and gave rise to several initiatives. Tools were designed to correct inconsistencies in the data [44,45,46]. Data was organized in publicly available databases, cross-referenced, and interoperable with the glycomic, and other omic, databases to ease data access and analysis, such as Glyco3D [47], UniLectin3D [48], and MatrixDB [42] for GAG-extracellular protein complexes. We now report the development of GAG-DB, a database containing three-dimensional data on GAGs and GAGs-protein complexes retrieved from the PDB. It includes protein sequences and standard nomenclature of GAG composition, sequence, and topology. It provides a family-based classification of GAGs, cross-referenced with glyco-databases, with links to UniProtKB via accession numbers [49]. The 3D visualization of contacts between GAGs and their protein ligands is implemented via the protein-ligand interaction profiler (PLIP) [50] and the nature of the structure that GAG polysaccharides can adopt, either in the solid-state or in solution is also reported. Finally, characterized quaternary structures of the complexes improve understanding if and how GAGs participate in long-range, multivalent, binding with the potential synergy when several chains are involved in interactions.

## 2. The GAG-DB Database Construction and Utilization

### 2.1. Database Construction 

GAG-DB is available at https://www.gagdb.glycopedia.eu. The database is populated with information extracted from the PDB [51]. It includes the three-dimensional structural information on GAG and GAG oligosaccharides in interaction with proteins. We propose a classification based on the nature of GAGs, e.g., hyaluronan, heparin/heparan sulfate, chondroitin sulfate/dermatan sulfate, and keratan sulfate. GAG mimetics are included, as long as they appear in the PDB. The content of GAG-DB is focused on three-dimensional data, with an appropriate curation of the nomenclature, and extended related information. The entries are structures of GAGs and GAG protein complexes obtained by a wide range of methods.

To avoid any confusion; we note that under the name GAG database, a resource to gather genomic annotation cross-references has been developed and published in 2013 (The GAG database: a new resource to gather genomic annotation cross-references, T Obadia, O Sallou, M Ouedraogo, G Guernec, F Lecerf and published (Gene. 2013;5;527(2):503-9., DOI:10.1016/j.gene.2013.06.063. Epub 16 2013 July). Available annotation data includes all transcripts and their identifiers, functional description of genes, chromosomal localisation, gene symbols, gene homologs for model species (human, chicken, mouse), and several identifiers to link those genes to external databases (UniProt, HGNC). 

The GAG-DB database contains 15 entries of long-chain GAGs established from fiber X-ray diffraction. A value of 3.0 Å is assigned to the structural models that have been proposed from X-ray fiber diffraction, and to 0 for those established by solution NMR or X-ray scattering (the structures are not filtered). It also contains 125 manually curated entries extracted from PDBe [52,53] (September 2020 release). These three-dimensional structures have been experimentally determined with methods involving either X-ray single-crystal diffraction, or X-ray fiber diffraction and solution NMR, in conjunction with molecular modeling. The number of GAG-protein complexes amounts to 105. The value of the resolution index indicates the accuracy of the experimental conditions, high values (e.g., 4 Å) indicate a poor resolution and low values (e.g., 1.5 Å) a good resolution. The median resolution for X-ray crystallographic data in the Protein Data Bank is 2.05 Å. Proteins of the database can be grossly separated into enzymes and skeletal proteins. Interestingly, the size distribution of oligosaccharides complexed with proteins varies from 34 disaccharides to only one polysaccharide with a degree of polymerization (DP) of 10 (DP 3 (1), DP 4 (18), DP 5 (13), DP 6 (15), DP 7 (7), DP 8 (8), and DP 9 (1). More than 80% of the GAGs involved in the complexes are heparin and hyaluronic acid oligosaccharides. However, these figures tend to reflect the interest of a community in investigating those GAGs more obviously involved in biological and biomedical applications.

Our collection is far from covering the molecular diversity of GAGs. This lack of data echoes the limitations of carbohydrate synthesis that fails to provide sufficiently long sequences needed to properly investigate the molecular features driving interactions with proteins. Nonetheless, progress is in sight, as recently described in [54,55].

The representation of GAGs sequences complies with recommended nomenclatures and formats, the IUPAC condensed being the reference (http://www.sbcs.qmul.ac.uk/iupac/2carb/38.html) [56]. Each sequence is also encoded in a machine-readable GlycoCT format [57,58], and depicted in SNFG (Symbol Nomenclature for Glycan) [22], following the description provided in [9].

At present, information associated with each entity of the database is added manually. This allows for proper curation and annotation, at the expense of a time lag between the date of deposition and the date of release in the database. Technically, the database was developed with PHP version 7, Bootstrap version 3 and MySQL database version 7. The interface is compatible with all devices and browsers. The pages are dynamically generated to match user-selected search criteria in the query window. Interactive graphics are developed in JavaScripts on D3JS libraries version 3. A tutorial is available on the first page.

### 2.2. Description of the Search Interface

The database can be searched and explored with an advanced search tool handling a range of criteria.

**Figure 2:**
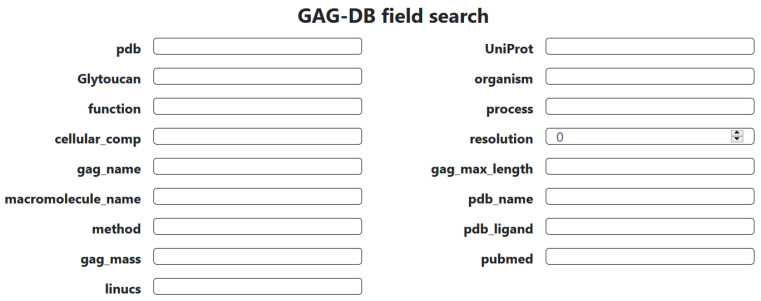
Multiple criteria of the advanced search in the GAG DB database.

Figure 2 shows the different fields that can be searched. Possible inputs are:

- The name of the polysaccharide **gag_name**, or its protein ligand, **macromolecule_name**.
- Cross-entries with external databases, such as **pdb**, **UniProt**, and repository **GlytouCan**.
- The biological role, such as **function**, **process**, or **cellular compartment** (compliant with Gene Ontology terms).
- The origin such as **organism**.
- The experimental condition(s) used to solve the structure: **method and resolution**.
- Characteristics of the GAG such as nature, (is_gag differentiates GAG and mimetics) and size (**gag_max, gag-length,** and **gag-mass**).
- Codes used for ligands in the Protein DataBase, **pdb_ligand** (nomenclature of the Chemical Component Dictionary. www.ebi.ac.uk/pdbe-srv/pdbechem/), or as encoded in **LINUCS** (Linear Notation for Unique description of Carbohydrate Sequences [58]), which provides access to **WURCS** (Web3 Unique Representation of Carbohydrate Structures [59]).

### 2.3. Curated Information for Each GAG Entry

**Figure 3:**
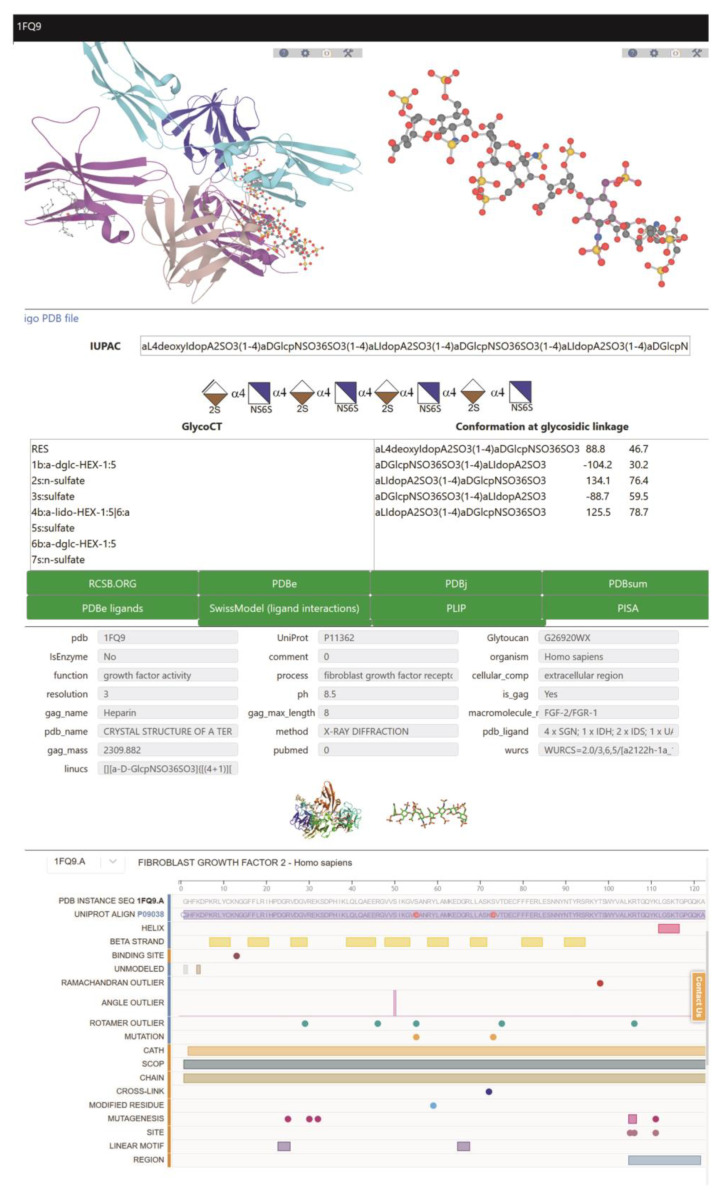
Full results from the search on GAG oligosaccharide present in the database using 1FQ9 in the PDB field. More information can be obtained by clicking on the green bars.

For each entry, a detailed page is available, with 3D visualization, interactions, conformations, nomenclature, and links to external databases (Figure 3). The PDB code assigned to each entry is used to list alternative structures and to display additional information. Each structure is related to a protein with a UniProt accession number [49]. Each oligosaccharide is given a GlyTouCan identifier [60]. The 3D structures of the protein and the interacting GAG are visualized directly and interactively with LiteMol [61] and NGL Viewers [62]. High-resolution images of both the protein-GAG complex and the GAG are available for download. The atomic coordinates of the GAG, isolated from its interaction with the proteins, can also be downloaded for further use. 

**Figure 4:**
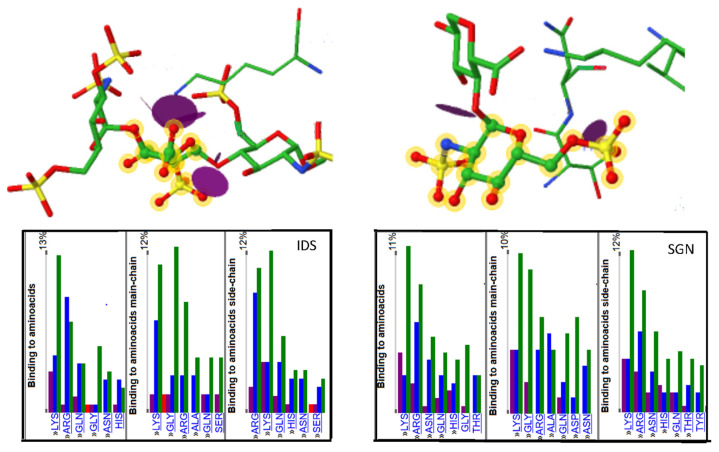
Example of graphical representation of GAG binding site obtained from the PDBe ligand interface. Distribution of the amino acids involved in the binding to GAGs oligosaccharides in cocrystallized complexes. Illustration of two cases showing the hydrogen bonding and electrostatic interaction involved in the interaction. https://www.ebi.ac.uk/pdbe-site/pdbemotif/?tab=ligandbindingstats&ligandCode3letter1=IDS; https://www.ebi.ac.uk/pdbe-site/pdbemotif/?tab=ligandbindingstats&ligandCode3letter1=SGN.

GAG-DB cross-references to several other databases that rely on a variety of strategies for visualizing the interaction between the GAG ligand and its protein environment (Figure 4). Several applications are available through the four different PDB sites, RSCG ORG, PDBe [51,53], PDBj [63], and PDB SUM [64].

Additional information on the interactions formed between the GAG and the protein can also be obtained using the protein-ligand interaction profiler (PLIP) server [50]. The NGL viewer [62] adapted to SwissModel [65] displays the interactions identified by the PLIP application that calculates and displays atomic level interactions (hydrogen bonds, hydrophobic, water bridge, etc.) occurring between GAGs and proteins. The specific features of the glycans interacting with the surrounding amino acid residues and possible metal ions are shown in 3D. The SwissModel application [65] provides direct access to the PDBsum deployed by the EMBL-EBI [64], CATH [66], and PLIP [50].

A cross-link to the PISA application [67,68] enables the exploration of quaternary structure formation and stability. The potential contribution of GAGs to the formation of quaternary macromolecular complexes requires the evaluation of energetic stability. The structural information relates to the interfaces between the macromolecular entities, the individual monomers, and the resulting assemblies, from which complex stability can be assessed or predicted. Supplementary Figures S1 and S2 provide examples of the interaction features offered by several visualization applications. 

## 3. Utilization of GAG-DB for Analysis of GAGs Structure and Conformation

### 3.1. Monosaccharides

Repeated disaccharide units of glycosamine and uronic acids with a non-uniform distribution of sulfated and acetylated groups along the chain constitute the main structural features of sulfated GAGs. Despite the high diversity of potential structures, only 28 unique monosaccharide structures occur in GAGs. Three of them correspond to 4,5 unsaturated uronic acids resulting from the eliminative cleavage of GAGs oligo- or polysaccharides containing (1->4)-linked d-glucuronate or l-iduronate residues and (1->4)-alpha-linked 2-sulfoamino-2-deoxy-6-sulfo-d-glucose residues to give oligosaccharides with terminal 4-deoxy-alpha-d-gluco-4-enuronosyl groups at their non-reducing ends.

The cartoon representation of monosaccharides was extended [42] in compliance with the SNFG representation of glycans [22] to link this description with the GlycoCT [58] and condensed IUPAC [56] codes of the monosaccharides.

**Figure 5:**
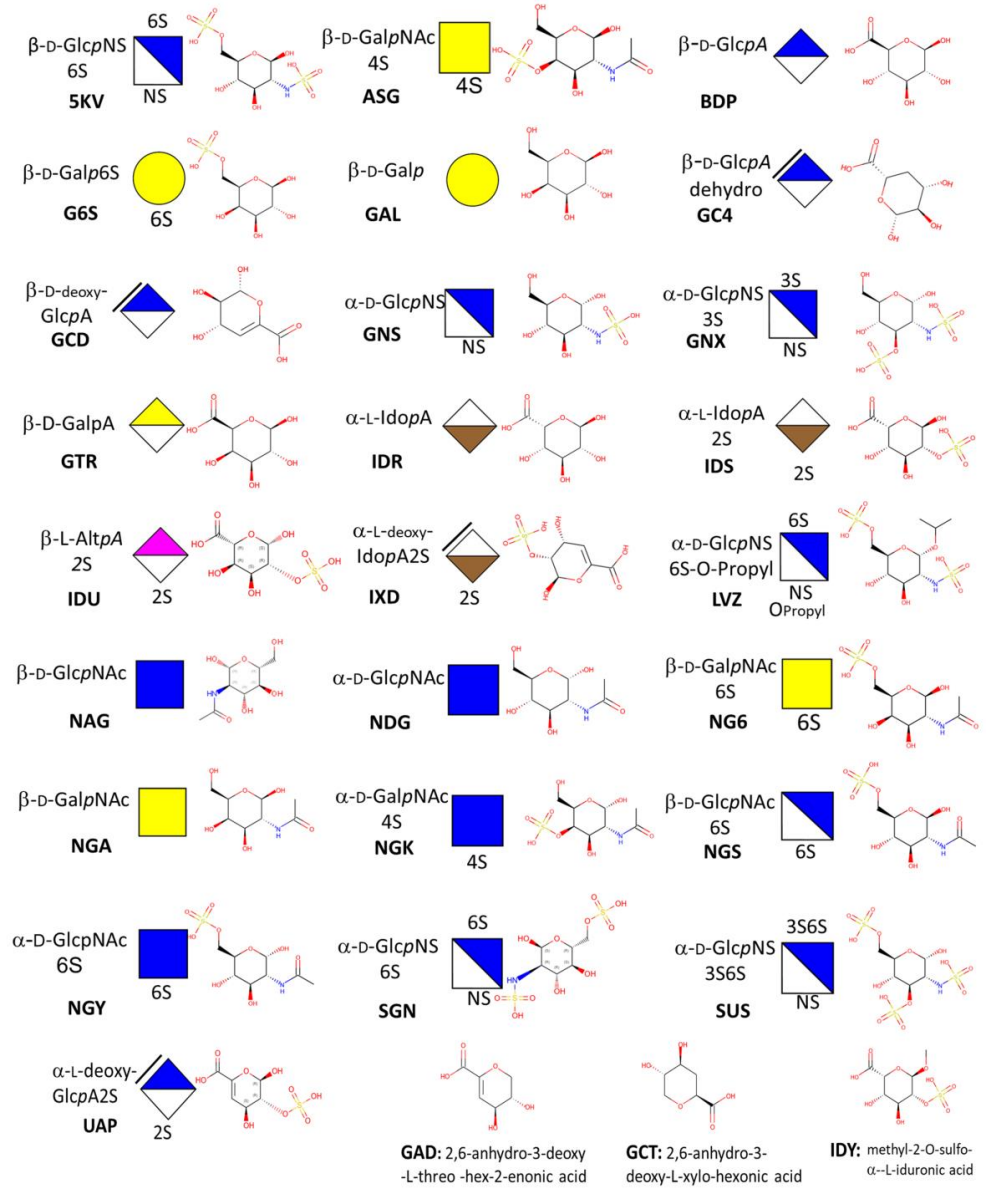
Cross-references between the common representations and nomenclature of monosaccharides: condensed IUAPC nomenclature, symbol nomenclature for glycan; PDB Chemical Component Nomenclature, 2-dimensional structure.

While these nomenclatures have become widely popular in the field of glycoscience, they are not used to identify and describe monosaccharides in the PDB, which has its carbohydrate nomenclature in its ligand dictionary [69]. Therefore, we established the cross-references between some of these nomenclatures (Figure 5).

**Figure 6:**
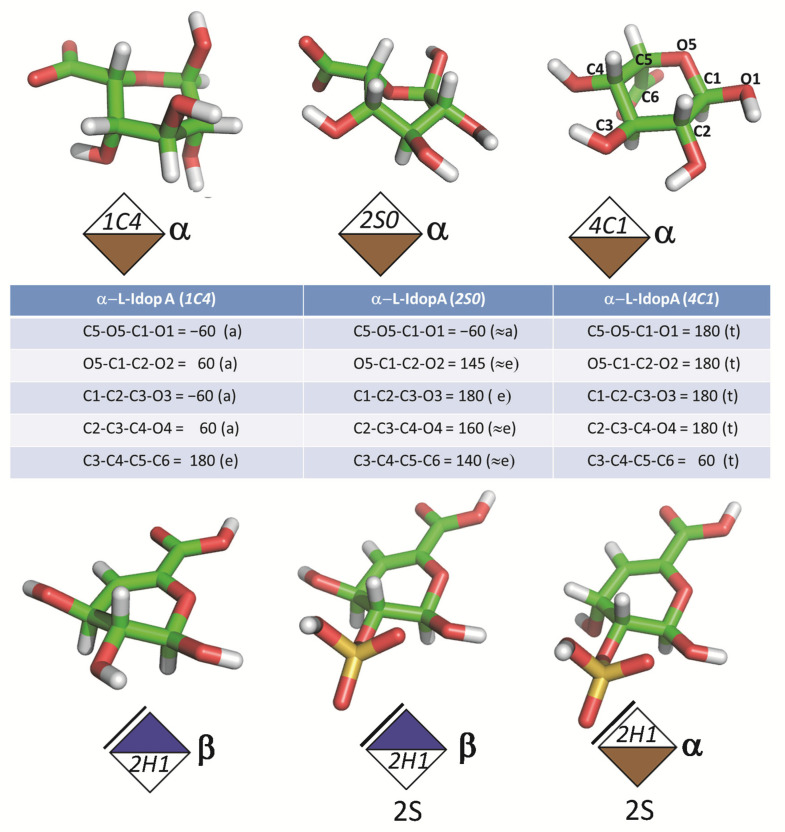
3-D structures, descriptors, and schematic representations of L-Idopyranosides, the following ^1^C_4_, ^4^C_1_, and ^2^S_0_; and 4,5 unsaturated uronic acids (drawn with pyMol (Schrödinger)).

Except for l-idopyranosides, and the 4,5 unsaturated uronic acids, all monosaccharides exist as hexopyranosides. The predominant conformation being ^4^C_1_. As for l-idopyranosides, the following ^1^C_4_, ^4^C_1_, and ^2^S_0_ conformations may be found. Figure 6 depicts the 3-dimensional representations of these unusual conformations, along with the corresponding SNFG extensions. 

### 3.2. Disaccharides

The PDB dataset consisting of 105 proteins-GAG complexes contains 270 disaccharides [9]. Table 1 displays the major disaccharides as extracted from GAG-DB.

**Figure 7:**
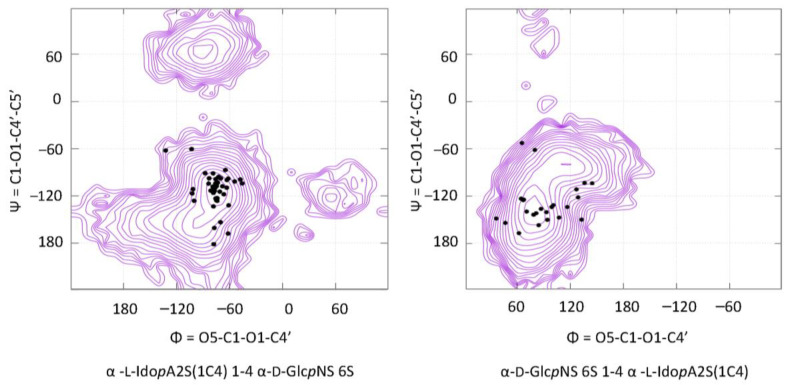
Φ and Ψ angles measured in the 3D structures of cocrystallized GAG protein complexes, reported on conformational maps.

Such a rich set of experimental data provides useful information to validate and improve computational strategies to build GAG models. The determination of the conformational preferences of GAG disaccharides can be assessed by computing potential energy surfaces as a function of their glycosidic torsion angles Φ and Ψ as implemented in the CAT application [70]. As an example, Figure 7 displays two such potential energy surfaces (alternatives are not shown). In all cases, the experimentally observed Φ and Ψ are plotted on the corresponding potential energy surfaces. While being somehow scattered, they are all located on the lowest energy basins. 

Similar features are observed for all disaccharides (or disaccharide units) irrespective of the presence and the positions of sulfate groups on the monosaccharides. The agreement between the repertoire of the experimentally determined conformations and those predicted by computational methods provided the basis to develop a pipeline to translate glycosaminoglycans sequences into 3D models (http://glycan-builder.cermav.cnrs.fr/gag/) [47]. 

### 3.3. GAG Structures in the Solid-State

The solid-state features of chondroitin sulfate, dermatan sulfate, hyaluronan, and keratan sulfate have been established by X-ray fiber diffraction [71] (Table 2) and are available in GAG-DB. They encompass several allomorphs that occur in different experimental conditions, including the nature of the counterions (Na^+^, Ca^++^, and K^+^). More structural features such as the polarity of the polysaccharide chains, their interactions with the counterions and packing features can be deduced.

The organization of all these polysaccharide chains in the form of helices seems recurrent. Two parameters, ***n*** and ***h***, characterize helical structures, where ***n*** is the number of repeat units (disaccharide unit) per turn of the helix and ***h*** is the projection of one repeat unit on the helical axis. The sign attributed to ***n*** indicates the chirality of the helix. The positive value of ***n*** corresponds to the right-handed helix and a negative value to a left-handed helix. Such helical descriptors provide a simple way to classify the secondary structures and their potential allomorphs.

**Figure 8:**
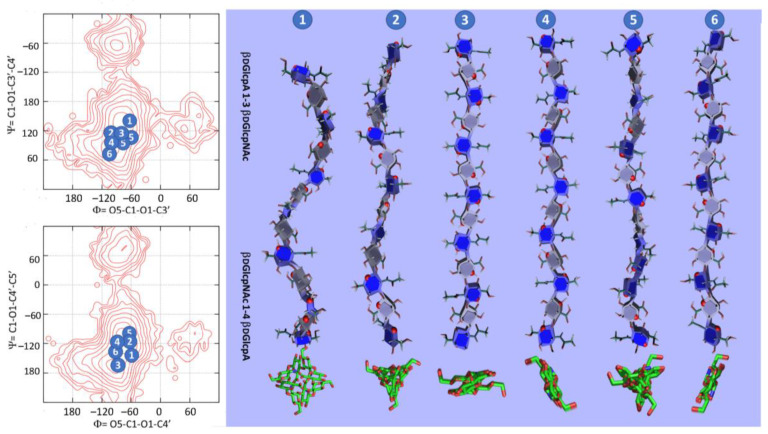
Stable regular helical conformation of single-stranded hyaluronic acid projected parallel and orthogonal to their axes (drawn with SweetUnityMol [82]). The Φ and Ψ conformations are shown on the corresponding potential energy surfaces.

As with the disaccharide segments of GAGs, the values of the Φ and Ψ torsional angles found in all the conformations of GAGs fall in the low energy regions of the corresponding potential energy surfaces. It is therefore relevant to question whether secondary structures other than those derived from crystallographic characterization do occur. The sets of (Φ, Ψ) values corresponding to the low energy conformations can be propagated regularly, to generate structures, which can be further optimized to form integral helices. When applied to hyaluronan structures, the analysis indicates that this polysaccharide display a wide range of energetically stable helices (Figure 8). They span the left-handed 4-fold symmetry to the right-handed five-fold symmetry with a rise per disaccharide between 9.51 and 10.13 Å [81].

The results indicate that small variations in the glycosidic torsion angles might have a significant influence on the symmetry and pitch of the resulting helices without any noticeable energetic cost. This illustrates the capacity of hyaluronic acid to display different sites available for interactions with proteins and would occur, at no cost in energy, without altering the directionality of the polysaccharide chain.

### 3.4. GAG Structures in Solution

**Figure 9:**
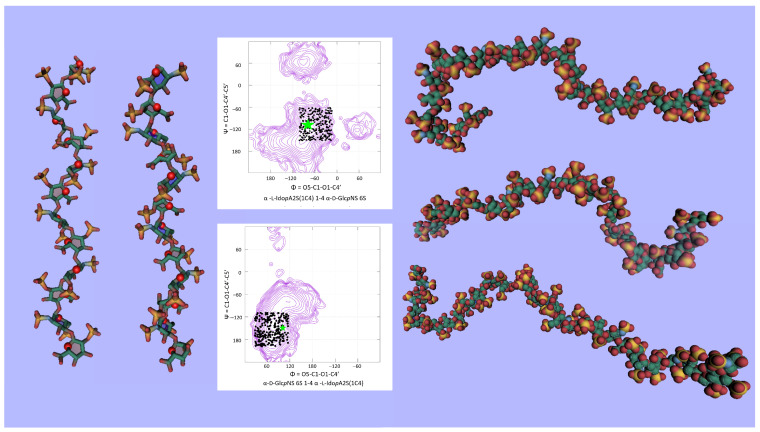
3D representation of heparin in a helical conformation (left panel) and in disordered conformation. The distribution of the Φ and Ψ angles are reported on the two corresponding potential energy surfaces (drawn with SweetUnityMol [82]).

The database contains the structure of heparin as established by NMR in solution (PDB entry 1HPN, 1XT3) and analogue (2ERM). Other structures have been reported for the solution structures of four different heparin oligosaccharides, determined by a combination of analytical ultracentrifugation, synchrotron X-ray solution scattering that gave the radii of gyration and maximum length extension [30,83] (PDB code 3IRI, 3IRJ, 3IRK, and 3IRL). Constrained molecular modeling of randomized heparin conformers resulted in 9–15 best-fit structures for each degree of polymerization (dp) DP18, DP24, DP30, and DP36 that indicated flexibility and the presence of short linear segments in mildly bent structures. All the conformations of the experimental conformations are somewhat scattered. They are all located in the lowest energy region of the corresponding Φ and Ψ maps (see Figure 9). The idopyranose residues experienced some changes, either ^1^C_4_ or ^2^S_0_, without any influence on the Φ and Ψ maps. This establishes a model of heparin in solution as a semi-rigid object.

Such a computational protocol was used to model the disordered features of hyaluronic acid [81] and chondroitin sulfate [84]. As with heparin, the semi-rigid behavior and the stiffness of these GAGs polysaccharides could be established. 

## 4. Conclusions

The aim of the article was to integrate three-dimensional data of GAGs, GAGs oligosaccharides as complexed with proteins. The sources of data are multiple: X-ray fiber diffraction, solution NMR, small angle X-ray scattering for GAGs, and X-ray biomolecular crystallography for protein-GAGs and protein-GAG mimetics complexes. A series of descriptors were selected to guide the search. They include cross-references to PDB, UniProtKB, MatrixDB, and GlyTouCan. GAG-DB opens the possibility of deciphering the full potential of GAGs as bioactive fragments or a structurally important multivalent scaffold for interaction synergy at assembling proteins within quaternary structures. The inspection of the many features of the database supports the reporting of robust facts/knowledge and the determination of what remains to be investigated or discovered. The amount and the quality of the 3D structures of GAG-protein complexes are amenable to comparison between the observed and the calculated 3D descriptors. Such a rich set of experimental information provides a solid basis for validating and improving computational strategies. We could confirm previously described features such as the lack of counterion effect in the interaction between GAGs; the definition of the preferred amino acids bringing the electrostatic neutrality of the interaction; and the lack of influence of sulfate groups on the glycosidic torsion angles. All the observed conformations fell within the low energy basins, thereby comforting the suitability of the computational protocol to model GAGs conformation in a disordered state. An emerging picture is the description of these polysaccharide chains as propagating linearly in a preferred direction, with extended fragments separated by kinks. The semi-rigid character of the chains involves microarchitectural domains. They contain preformed conformation for optimal binding to protein targets. The separation of such domains, at a long enough distance, offers the possibility of multivalent binding to create further spatial arrangements that can induce the formation of functional assemblies of proteins.

## Figures and Tables

**Table 1:** Major disaccharides found in the GAG-protein complexes.

Major Disaccharides Found in the GAG-Protein Complexes Extracted from the PDB	Number
α-d-Glc*p*NS (1-4) β-d-Glc*p*A	21
α-d-Glc*p*NS(6S) (1-4) α-l-Ido*p*A(2S) [1C4]	42
α-d-Glc*p*NS(6S) (1-4) α-l-Ido*p*A(2S) [2S0]	15
α-l-Ido*p*A(2S) [1C4] (1-4) α-d-Glc*p*NS(6S)	40
α-l-Ido*p*A(2S) [2S0] (1-4) α-d-Glc*p*NS(6S)	16
β-d-Glc*p*A (1-3) β-d-Glc*p*NAc	13
β-d-Glc*p*A (1-4) α-d-Glc*p*NS	15
β-d-Glc*p*NAc (1-4) β-d-Glc*p*A	12

**Table 2:** Characterization of the helix symmetry of GAGs polysaccharides in the solid-state.

Glycosaminoglycans	Structure of the Main Repeating Disaccharides	Helix Symmetry	Ref.
Hyaluronan	-4)-β-d-Glc*p*A-(1-3)-β-d-Glc*p*NAc-(1-	2_1_, 3_2_, 4_3_	[72,73,74]
Chondroitin-4-sulfate	-4)-β-d-Glc*p*A-(1-3)-β-d-Gal*p*NAc(4S)-(1-	2_1_, 3_2_	[75,76,77]
Chondroitin-6-sulfate	-4)-β-d-Glc*p*A-(1-3)-β-d-Gal*p*NAc(6S)-(1-	2_1_, 3_2_, 8_3_	
Dermatan sulfate	-4)-α-l-Ido*p*A-(1-3)-β-d-Gal*p*NAc(4S)-(1-	2_1_, 3_2_, 8_3_	[78]
Heparin	-4)-α-l-Ido*p*A(2S)-(1-4)-α-d-Glc*p*NS(6S)-(1	(Na^+^) 2_1_	
Heparan sulfate	-4)-β-d-Glc*p*A-(1-4)-α-d-Glc*p*NAc-(1-	2_1_	[79]
Keratan sulfate	-3)-β-d-Gal*p*-(1-4)-β-d-Glc*p*NAc(6S)-(1-	2_1_	[80]

## Supplementary Materials

The following are available online at https://www.mdpi.com/2218-273X/10/12/1660/s1, Figure S1: Detailed of the analysis of GAG binding in crystal structure of a ternary FGF1-FGFR2-Heparin complex (PDB 2GD4) available from different souces; PDBe; Swiss-Model; PLIP, LIGPOLT. Figure S2: Quaternary organisation in the crystal Structure of the Antithrombin-S195A Factor Xa-Pentasaccharide Complex (PDB 1EO0) computed and displayed by PISA.

## References

[1] Iozzo R.V., Schaefer L. (2015). Proteoglycan form and function: A comprehensive nomenclature of proteoglycans. Matrix Biol..

[2] Karamanos N.K., Piperigkou Z., Theocharis A.D., Watanabe H., Franchi M., Baud S., Brezillon S., Gotte M., Passi A., Vigetti D. (2018). Proteoglycan chemical diversity drives multifunctional cell regulation and therapeutics. Chem. Rev..

[3] Mikami T., Kitagawa H. (2013). Biosynthesis and function of chondroitin sulfate. Biochim. Biophys. Acta.

[4] Gallagher J. (2015). Fell-Muir Lecture: Heparan sulphate and the art of cell regulation: A polymer chain conducts the protein orchestra. Int. J. Exp. Pathol..

[5] Li J.P., Kusche-Gullberg M. (2016). Heparan sulfate: Biosynthesis, structure, and function. Int. Rev. Cell. Mol. Biol..

[6] Garantziotis S., Savani R.C. (2019). Hyaluronan biology: A complex balancing act of structure, function, location and context. Matrix Biol..

[7] Caterson B., Melrose J. (2018). Keratan sulfate, a complex glycosaminoglycan with unique functional capability. Glycobiology.

[8] Pomin V.H. (2015). Keratan sulfate: An up-to-date review. Int. J. Biol. Macromol..

[9] Clerc O., Mariethoz J., Rivet A., Lisacek F., Perez S., Ricard-Blum S. (2019). A pipeline to translate glycosaminoglycan sequences into 3D models. Application to the exploration of glycosaminoglycan conformational space. Glycobiology.

[10] Vallet S.D., Clerc O., Ricard-Blum S. (2020). Glycosaminoglycan-protein Interactions: The first draft of the glycosaminoglycan interactome. J. Histochem. Cytochem..

[11] Peysselon F., Ricard-Blum S. (2014). Heparin-protein interactions: From affinity and kinetics to biological roles. Application to an interaction network regulating angiogenesis. Matrix Biol..

[12] Ricard-Blum S. (2017). Glycosaminoglycans: Major biological players. Glycoconj. J..

[13] Ori A., Wilkinson M.C., Fernig D.G. (2011). A systems biology approach for the investigation of the heparin/heparan sulfate interactome. J. Biol. Chem..

[14] Ricard-Blum S., Lisacek F. (2017). Glycosaminoglycanomics: Where we are. Glycoconj. J..

[15] Aquino R.S., Park P.W. (2016). Glycosaminoglycans and infection. Front. Biosci..

[16] Burns J.M., Lewis G.K., DeVico A.L. (1999). Soluble complexes of regulated upon activation, normal T cells expressed and secreted (RANTES) and glycosaminoglycans suppress HIV-1 infection but do not induce Ca(2+) signaling. Proc. Natl. Acad. Sci. USA.

[17] Fatoux-Ardore M., Peysselon F., Weiss A., Bastien P., Pratlong F., Ricard-Blum S. (2014). Large-scale investigation of Leishmania interaction networks with host extracellular matrix by surface plasmon resonance imaging. Infect. Immun..

[18] Hsiao F.S., Sutandy F.R., Syu G.D., Chen Y.W., Lin J.M., Chen C.S. (2016). Systematic protein interactome analysis of glycosaminoglycans revealed YcbS as a novel bacterial virulence factor. Sci. Rep..

[19] Jinno A., Park P.W. (2015). Role of glycosaminoglycans in infectious disease. Methods Mol. Biol..

[20] Merida-de-Barros D.A., Chaves S.P., Belmiro C.L.R., Wanderley J.L.M. (2018). Leishmaniasis and glycosaminoglycans: A future therapeutic strategy?. Parasit. Vect..

[21] Casu B., Naggi A., Torri G. (2015). Re-visiting the structure of heparin. Carbohydr. Res..

[22] Varki A., Cummings R.D., Aebi M., Packer N.H., Seeberger P.H., Esko J.D., Stanley P., Hart G., Darvill A., Kinoshita T. (2015). Symbol nomenclature for graphical representations of glycans. Glycobiology.

[23] Silva J.C., Carvalho M.S., Han X., Xia K., Mikael P.E., Cabral J.M.S., Ferreira F.C., Linhardt R.J. (2019). Compositional and structural analysis of glycosaminoglycans in cell-derived extracellular matrices. Glycoconj. J..

[24] Volpi N., Linhardt R.J. (2010). High-performance liquid chromatography-mass spectrometry for mapping and sequencing glycosaminoglycan-derived oligosaccharides. Nat. Protoc..

[25] Wu J., Wei J., Chopra P., Boons G.J., Lin C., Zaia J. (2019). Sequencing heparan sulfate using HILIC LC-NETD-MS/MS. Anal. Chem..

[26] Yu Y., Duan J., Leach F.E., Toida T., Higashi K., Zhang H., Zhang F., Amster I.J., Linhardt R.J. (2017). Sequencing the Dermatan Sulfate Chain of Decorin. J. Am. Chem. Soc..

[27] Zaia J. (2013). Glycosaminoglycan glycomics using mass spectrometry. Mol. Cell Proteom..

[28] Langeslay D.J., Beni S., Larive C.K. (2011). Detection of the 1H and 15N NMR resonances of sulfamate groups in aqueous solution: A new tool for heparin and heparan sulfate characterization. Anal. Chem..

[29] Pomin V.H. (2016). (1)H and (15)N NMR analyses on heparin, heparan sulfates and related monosaccharides concerning the chemical exchange regime of the N-sulfo-glucosamine sulfamate proton. Pharmaceuticals.

[30] Khan S., Fung K.W., Rodriguez E., Patel R., Gor J., Mulloy B., Perkins S.J. (2013). The solution structure of heparan sulfate differs from that of heparin: Implications for function. J. Biol. Chem..

[31] Jasnin M. (2012). Use of neutrons reveals the dynamics of cell surface glycosaminoglycans. Methods Mol. Biol..

[32] Rubinson K.A., Chen Y., Cress B.F., Zhang F., Linhardt R.J. (2016). Heparin’s solution structure determined by small-angle neutron scattering. Biopolymers.

[33] Scherbinina S.I., Toukach P.V. (2020). Three-dimensional structures of carbohydrates and where to find them. Intern. J. Mol. Sci..

[34] Samsonov S.A., Pisabarro M.T. (2016). Computational analysis of interactions in structurally available protein-glycosaminoglycan complexes. Glycobiology.

[35] Sankaranarayanan N.V., Nagarajan B., Desai U.R. (2018). So you think computational approaches to understanding glycosaminoglycan-protein interactions are too dry and too rigid? Think again!. Curr. Opin. Struct. Biol..

[36] Sattelle B.M., Almond A. (2014). Microsecond kinetics in model single- and double-stranded amylose polymers. Phys. Chem. Chem. Phys..

[37] Sattelle B.M., Shakeri J., Cliff M.J., Almond A. (2015). Proteoglycans and their heterogeneous glycosaminoglycans at the atomic scale. Biomacromolecules.

[38] Almond A. (2018). Multiscale modeling of glycosaminoglycan structure and dynamics: Current methods and challenges. Curr. Opin. Struct. Biol..

[39] Kolesnikov A.L., Budkov Y.A., Nogovitsyn E.A. (2014). Coarse-grained model of glycosaminoglycans in aqueous salt solutions. A field-theoretical approach. J. Phys. Chem. B.

[40] Samsonov S.A., Bichmann L., Pisabarro M.T. (2015). Coarse-grained model of glycosaminoglycans. J. Chem. Inf. Model..

[41] Whitmore E.K., Martin D., Guvench O. (2020). Constructing 3-dimensional atomic-resolution models of nonsulfated glycosaminoglycans with arbitrary lengths using conformations from molecular dynamics. Intern. J. Mol. Sci..

[42] Clerc O., Deniaud M., Vallet S.D., Naba A., Rivet A., Perez S., Thierry-Mieg N., Ricard-Blum S. (2019). MatrixDB: Integration of new data with a focus on glycosaminoglycan interactions. Nucleic Acids Res..

[43] Berman H.M., Westbrook J., Feng Z., Gilliland G., Bhat T.N., Weissig H., Shindyalov I.N., Bourne P.E. (2000). The Protein Data Bank. Nucleic Acids Res..

[44] Bagdonas H., Ungar D., Agirre J. (2020). Leveraging glycomics data in glycoprotein 3D structure validation with Privateer. Beilstein J. Org. Chem..

[45] Lutteke T., von der Lieth C.W. (2004). pdb-care (PDB carbohydrate residue check): A program to support annotation of complex carbohydrate structures in PDB files. BMC Bioinform..

[46] Sehnal D., Svobodova Varekova R., Pravda L., Ionescu C.M., Geidl S., Horsky V., Jaiswal D., Wimmerova M., Koca J. (2015). ValidatorDB: Database of up-to-date validation results for ligands and non-standard residues from the Protein Data Bank. Nucleic Acids Res..

[47] Perez S., Sarkar A., Rivet A., Breton C., Imberty A. (2015). Glyco3D: A portal for structural glycosciences. Methods Mol. Biol..

[48] Bonnardel F., Mariethoz J., Salentin S., Robin X., Schroeder M., Perez S., Lisacek F., Imberty A. (2019). UniLectin3D, a database of carbohydrate binding proteins with curated information on 3D structures and interacting ligands. Nucleic Acids Res..

[49] UniProt C. (2019). UniProt: A worldwide hub of protein knowledge. Nucleic Acids Res..

[50] Salentin S., Schreiber S., Haupt V.J., Adasme M.F., Schroeder M. (2015). PLIP: Fully automated protein-ligand interaction profiler. Nucleic Acids Res..

[51] Mir S., Alhroub Y., Anyango S., Armstrong D.R., Berrisford J.M., Clark A.R., Conroy M.J., Dana J.M., Deshpande M., Gupta D. (2018). PDBe: Towards reusable data delivery infrastructure at protein data bank in Europe. Nucleic Acids Res..

[52] Gutmanas A., Alhroub Y., Battle G.M., Berrisford J.M., Bochet E., Conroy M.J., Dana J.M., Fernandez Montecelo M.A., van Ginkel G., Gore S.P. (2014). PDBe: Protein Data Bank in Europe. Nucleic Acids Res..

[53] PDBe-KB-Consortium (2020). PDBe-KB: A community-driven resource for structural and functional annotations. Nucleic Acids Res..

[54] Pardo-Vargas A., Delbianco M., Seeberger P.H. (2018). Automated glycan assembly as an enabling technology. Curr. Opin. Chem. Biol..

[55] Pomin V.H., Wang X. (2018). Synthetic oligosaccharide libraries and microarray technology: A powerful combination for the success of current glycosaminoglycan interactomics. ChemMedChem.

[56] McNaught A.D. (1997). Nomenclature of carbohydrates (recommendations 1996). Adv. Carbohydr. Chem. Biochem..

[57] Herget S., Ranzinger R., Maass K., Lieth C.W. (2008). GlycoCT-a unifying sequence format for carbohydrates. Carbohydr. Res..

[58] Lutteke T., Bohne-Lang A., Loss A., Goetz T., Frank M., von der Lieth C.W. (2006). GLYCOSCIENCES.de: An Internet portal to support glycomics and glycobiology research. Glycobiology.

[59] Tanaka K., Aoki-Kinoshita K.F., Kotera M., Sawaki H., Tsuchiya S., Fujita N., Shikanai T., Kato M., Kawano S., Yamada I. (2014). WURCS: The Web3 unique representation of carbohydrate structures. J. Chem. Inf. Model..

[60] Tiemeyer M., Aoki K., Paulson J., Cummings R.D., York W.S., Karlsson N.G., Lisacek F., Packer N.H., Campbell M.P., Aoki N.P. (2017). GlyTouCan: An accessible glycan structure repository. Glycobiology.

[61] Sehnal D., Deshpande M., Varekova R.S., Mir S., Berka K., Midlik A., Pravda L., Velankar S., Koca J. (2017). LiteMol suite: Interactive web-based visualization of large-scale macromolecular structure data. Nat. Methods.

[62] Rose A.S., Bradley A.R., Valasatava Y., Duarte J.M., Prlic A., Rose P.W. (2018). NGL viewer: Web-based molecular graphics for large complexes. Bioinformatics.

[63] Kinjo A.R., Bekker G.J., Wako H., Endo S., Tsuchiya Y., Sato H., Nishi H., Kinoshita K., Suzuki H., Kawabata T. (2018). New tools and functions in data-out activities at Protein Data Bank Japan (PDBj). Protein Sci..

[64] Laskowski R.A., Jablonska J., Pravda L., Varekova R.S., Thornton J.M. (2018). PDBsum: Structural summaries of PDB entries. Protein Sci..

[65] Waterhouse A., Bertoni M., Bienert S., Studer G., Tauriello G., Gumienny R., Heer F.T., de Beer T.A.P., Rempfer C., Bordoli L. (2018). SWISS-MODEL: Homology modelling of protein structures and complexes. Nucleic Acids Res..

[66] Sillitoe I., Dawson N., Lewis T.E., Das S., Lees J.G., Ashford P., Tolulope A., Scholes H.M., Senatorov I., Bujan A. (2019). CATH: Expanding the horizons of structure-based functional annotations for genome sequences. Nucleic Acids Res..

[67] Krissinel E. (2010). Crystal contacts as nature’s docking solutions. J. Comput. Chem..

[68] Krissinel E., Henrick K. (2007). Inference of macromolecular assemblies from crystalline state. J. Mol. Biol..

[69] Dimitropoulos D., Ionides J., Henrick K. (2006). Using MSDchem to search the PDB ligand dictionary. Curr. Protoc. Bioinform..

[70] Frank M., Lutteke T., von der Lieth C.W. (2007). GlycoMapsDB: A database of the accessible conformational space of glycosidic linkages. Nucleic Acids Res..

[71] Smith P.J.C., Arnott S. (1978). LALS, a linked-atom least-squares reciprocal-space refinement system incorporating stereochemical restraints to supplement sparse diffraction data. Acta Crystallogr. Sect. A Found. Crystallogr..

[72] Guss J.M., Hukins D.W., Smith P.J., Winter W.T., Arnott S. (1975). Hyaluronic acid: Molecular conformations and interactions in two sodium salts. J. Mol. Biol..

[73] Mitra A.K., Arnott S., Sheehan J.K. (1983). Hyaluronic acid: Molecular conformation and interactions in the tetragonal form of the potassium salt containing extended chains. J. Mol. Biol..

[74] Winter W.T., Arnott S. (1977). Hyaluronic acid: The role of divalent cations in conformation and packing. J. Mol. Biol..

[75] Cael J.J., Winter W.T., Arnott S. (1978). Calcium chondroitin 4-sulfate: Molecular conformation and organization of polysaccharide chains in a proteoglycan. J. Mol. Biol..

[76] Millane R.P., Mitra A.K., Arnott S. (1983). Chondroitin 4-sulfate: Comparison of the structures of the potassium and sodium salts. J. Mol. Biol..

[77] Winter W.T., Arnott S., Isaac D.H., Atkins E.D. (1978). Chondroitin 4-sulfate: The structure of a sulfated glycosaminoglycan. J. Mol. Biol..

[78] Mitra A.K., Arnott S., Atkins E.D., Isaac D.H. (1983). Dermatan sulfate: Molecular conformations and interactions in the condensed state. J. Mol. Biol..

[79] Lee S.C., Guan H.H., Wang C.H., Huang W.N., Tjong S.C., Chen C.J., Wu W.G. (2005). Structural basis of citrate-dependent and heparan sulfate-mediated cell surface retention of cobra cardiotoxin A3. J. Biol. Chem..

[80] Arnott S., Gus J.M., Hukins D.W., Dea I.C., Rees D.A. (1974). Conformation of keratan sulphate. J. Mol. Biol..

[81] Haxaire K., Braccini I., Milas M., Rinaudo M., Perez S. (2000). Conformational behavior of hyaluronan in relation to its physical properties as probed by molecular modeling. Glycobiology.

[82] Perez S., Tubiana T., Imberty A., Baaden M. (2015). Three-dimensional representations of complex carbohydrates and polysaccharides—SweetUnityMol: A video game-based computer graphic software. Glycobiology.

[83] Khan S., Gor J., Mulloy B., Perkins S.J. (2010). Semi-rigid solution structures of heparin by constrained X-ray scattering modelling: New insight into heparin-protein complexes. J. Mol. Biol..

[84] Rodriguez-Carvajal M.A., Imberty A., Perez S. (2003). Conformational behavior of chondroitin and chondroitin sulfate in relation to their physical properties as inferred by molecular modeling. Biopolymers.

