# Supplementary material for: GAG-DB, the New Interface of the Three-Dimensional Landscape of Glycosaminoglycans
Source: Biomolecules. 2020 Dec 11;10(12):1660. doi: 10.3390/biom10121660 (PMC7763844; doi:10.3390/biom10121660)
Supplement: Supplementary file 1 [file biomolecules-10-01660-s001.pdf]

Supplementary Figures

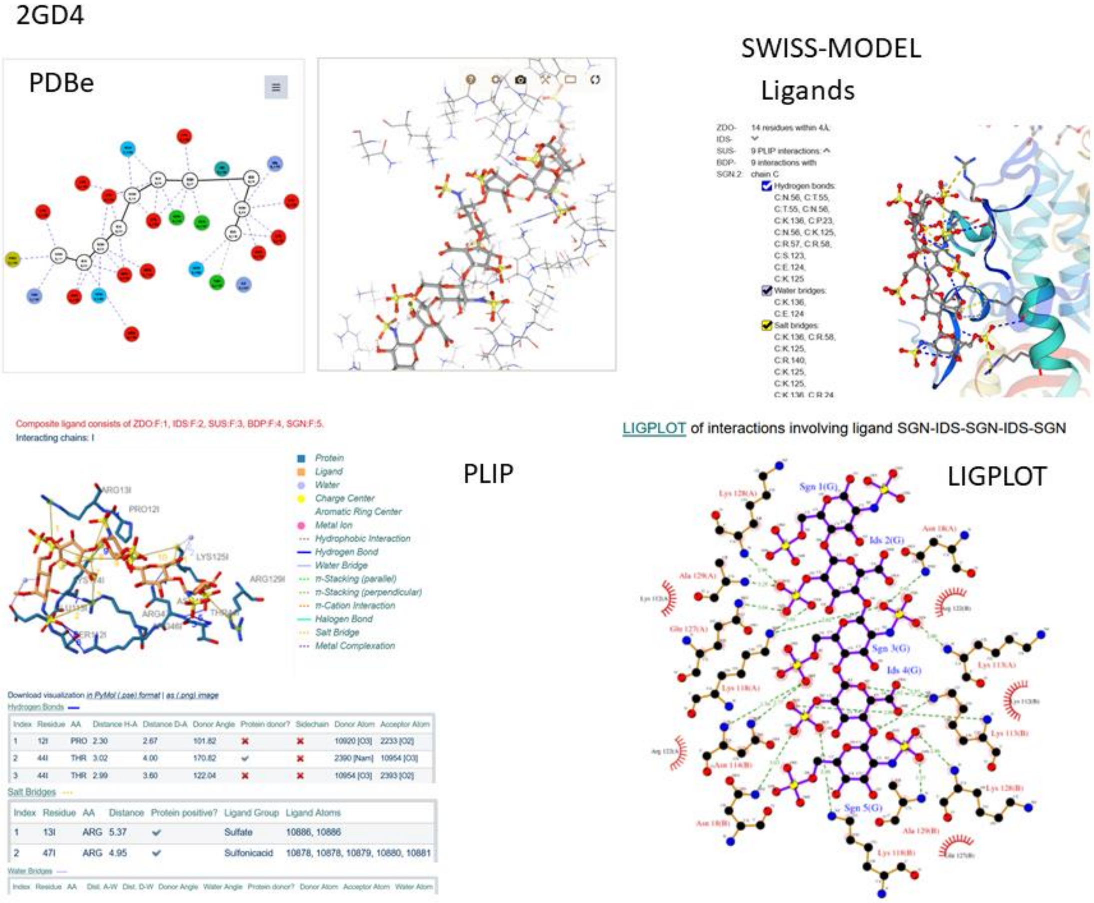

Figure S1. Detailed of the analysis of GAG binding in crystal structure of a ternary FGF1-FGFR2-Heparin complex (PDB 2GD4) available from different souces ; PDBe ; Swiss-Model ; PLIP, LIGPOLT.

1ᴱ0o

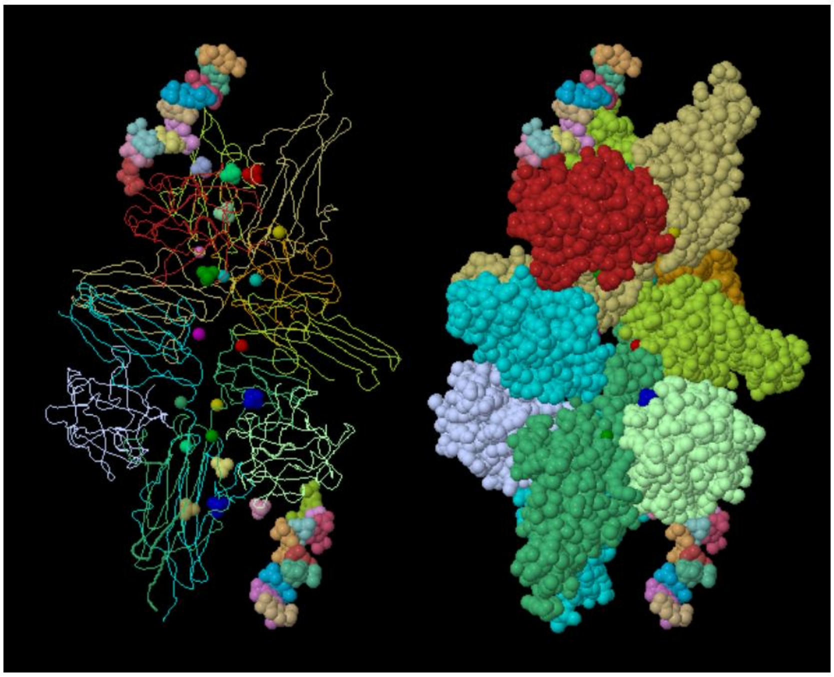

Figure S2. Quaternary organisation in the crystal Structure of the Antithrombin-S195A Factor Xa-Pentasaccharide Complex (PDB 1EO0) computed and displayed by PISA.
